# Radiation Protection Compliance in Fluoroscopy-Assisted Procedures: A Prospective Audit in Operating Theatres

**DOI:** 10.7759/cureus.99365

**Published:** 2025-12-16

**Authors:** Abdulshakor Ali, Kumaresan Balasundram, Sabina Gunzler

**Affiliations:** 1 Trauma and Orthopaedics, Oxford University Hospitals NHS Foundation Trust, Oxford, GBR; 2 Radiology, Oxford University Hospitals NHS Foundation Trust, Oxford, GBR

**Keywords:** education, hospitalist resident, major trauma, orthopaedic spine surgery, quality improvement, radiology audit, thyroid cancer, thyroid shield

## Abstract

Background: Fluoroscopy-assisted procedures expose operating theatre staff to ionising radiation, with the thyroid, eyes, and hands being particularly vulnerable. Despite established protection protocols, compliance remains inconsistent across clinical roles.

Objective: This study aims to evaluate adherence to radiation protection protocols, particularly thyroid shielding, among theatre staff during fluoroscopy-assisted procedures, identify compliance gaps, and propose targeted interventions.

Materials and methods: A prospective audit was conducted over 10 weeks (May to August 2025) in the operating theatres of a level 1 major trauma centre (MTC) in the United Kingdom. Observational data were collected on thyroid protection use, supplemented by dosimetry and adherence to personal protective equipment (PPE) checklists. A total of 712 (100%) staff members were observed during 91 fluoroscopy-guided procedures. Notably, the audit was conducted in a blinded manner; no theatre staff were aware that radiation protection behaviour was being monitored, apart from the radiographers. This approach ensured that the data reflected authentic staff conduct without being influenced by observational bias. Chi-square tests were used to assess differences in compliance across roles and specialities, with statistical significance set at p < 0.05.

Results: Among the 712 (100%) staff members assessed during 91 operations, 555 (78.0%) demonstrated full compliance with lead gown and thyroid protection protocols. However, 157 (22.0%) were non-compliant with thyroid protection. Of these, roughly 150 (21%) wore a lead gown without a thyroid shield, and seven (1.0%) wore neither a gown nor a thyroid shield.

When examining the distribution of non-compliant cases across operation types, 79.1% (n = 72) occurred during trauma and orthopaedic surgeries, 17.6% (n = 16) during spine surgeries, and 3.3% (n = 3) during other procedures. Chi-square analysis revealed no statistically significant association between operation type and PPE non-compliance (p = 0.0995, df = 2), exceeding the conventional threshold of 0.05.

When analysed by role, non-compliance was observed in 38.7% (n = 58) of surgical team members, 26.0% (n = 39) of scrub nurses, 20.7% (n = 31) of anaesthetic staff, and 14.7% (n = 22) of individuals in other roles. (χ² = 14.87, p < 0.001), with odds of non-compliance 2.31 times greater (OR = 2.31, 95% CI: 1.52-3.52). Scrub nurses also demonstrated elevated non-compliance (χ² = 7.43, p = 0.006, OR = 1.74, 95% CI: 1.18-2.89), as did anaesthetic staff (χ² = 5.12, p = 0.024, OR = 1.58, 95% CI: 1.06-2.52) and individuals from other roles (χ² = 3.87, p = 0.049, OR = 1.42, 95% CI: 1.01-2.21).

Conclusions: Although overall adherence to thyroid protection protocols was high, notable and statistically significant gaps were identified, particularly among trauma surgeons, scrub nurses, and anaesthetic staff. These findings underscore the need for targeted educational initiatives, routine equipment audits, and ongoing compliance monitoring to strengthen radiation safety practices across all clinical roles.

## Introduction

Fluoroscopy-assisted procedures are integral to modern surgical and interventional practice, particularly in orthopaedics and spinal surgery, where real-time imaging enhances precision and improves clinical outcomes. However, these procedures expose theatre staff to ionising radiation, which poses long-term health risks, including thyroid dysfunction, cataracts, and increased cancer risk [1-3]. The thyroid gland, eyes, and hands are especially vulnerable to cumulative exposure, making the use of personal protective equipment (PPE), such as thyroid shields and lead gowns, essential for mitigating these risks [4].

Radiation dose quantifies the energy deposited by ionising particles and is calculated as the product of exposure and fluoroscopy time. The absorbed dose Gray (Gy) reflects the physical energy absorbed per kilogram of tissue. In contrast, the equivalent dose in Sieverts (Sv) adjusts for radiation type using a weighting factor (Wr), which is 1 for X-rays. The effective dose (Sv) further refines this by summing the equivalent doses across organs, each multiplied by a tissue weighting factor, to estimate overall biological risk [5]. The thyroid gland, with a tissue weighting factor of 0.04, is susceptible to radiation. For example, an absorbed dose of 0.142 millisieverts (mSv) to the thyroid corresponds to an effective dose of 0.00568 mSv. According to the International Commission on Radiological Protection (ICRP) guidelines, the occupational practical dose limit is 20 mSv per year, averaged over five years, with no single year exceeding 50 mSv [5].

In orthopaedic procedures such as femoral intramedullary (IM) nailing, the effective dose varies with projection and technique, typically ranging from 1 to 3 mSv [6]. Using dose-area product values, which represent the total radiation energy delivered, the effective dose can be estimated as approximately 0.85 mSv for 500 centigray times square centimetres (cGy·cm²) and 2.55 millisieverts (mSv) for 1500 cGy·cm² [7]. Factors influencing radiation dose include fracture complexity (which dictates the required imaging, AP vs. lateral views), implant type, surgeon experience, and patient size. More complex fractures and longer implants require longer imaging time, while less-experienced surgeons may rely more heavily on fluoroscopy. Larger patients also require higher radiation doses to achieve adequate image clarity [5,8].

Despite clear guidelines from professional bodies such as the ICRP and the Royal College of Radiologists, compliance with radiation safety protocols remains variable [4,9]. This audit was conducted to evaluate current adherence to radiation protection standards in the operating theatre, a level 1 major trauma centre (MTC) in the United Kingdom, with a particular focus on thyroid shielding during fluoroscopy-assisted procedures.

## Materials and methods

Setting and duration

The audit was conducted in the main operating theatres of an MTC in the United Kingdom between 27 May and 6 August 2025.

Design

This was a prospective observational audit of practice. Data collection methods included direct observations of PPE use during fluoroscopy-guided procedures, dosimetry to assess exposure risk to sensitive anatomical sites, and checklist assessments to evaluate adherence to PPE protocols.

The inclusion criteria encompassed all staff present during fluoroscopy-assisted procedures and procedures that involved real-time imaging guidance. Exclusion criteria included procedures without fluoroscopy and staff members not directly involved in the operative field.

Statistical analysis

Descriptive statistics were used to calculate compliance rates. Chi-square tests (χ²) were applied to compare compliance across roles and specialties. Odds ratios (OR) and 95% confidence intervals (CI) were calculated to assess the strength of association. A p-value < 0.05 was considered statistically significant.

## Results

Overall compliance

A total of 712 (100%) staff members were observed during fluoroscopy-assisted procedures. Among them, 555 (78.0%) were fully compliant with thyroid protection protocols. Non-compliance with thyroid protection was identified in 150 (21.1%) cases, and seven (1.0%) were observed without any thyroid protection or a radiation gown.

Breakdown by specialty operations

**Figure 1:**
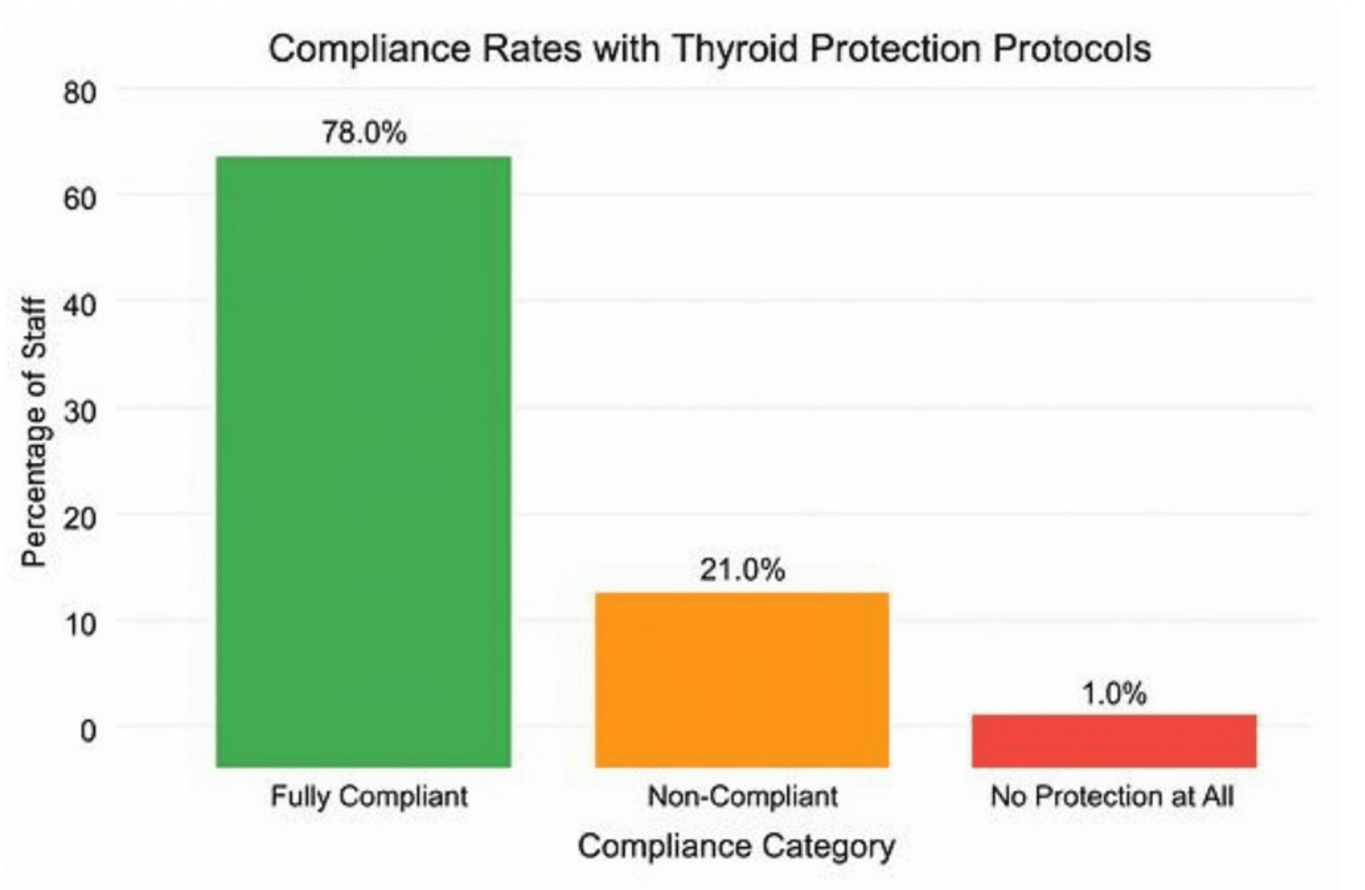
Percentage of staff members adherence to thyroid protection protocols

Among the 150 non-compliant (no thyroid shields) individuals reviewed, trauma and orthopaedic operations accounted for the highest proportion of non-adherence to thyroid protection guidelines, with 72 cases (comprising 55 adult and 17 paediatric procedures), representing 79.1% of the total 91 cases. Spine surgeries contributed 16 (17.6%) cases out of 91 cases, while other procedures accounted for three (3.3%) cases. A chi-square test revealed a statistically significant difference in non-compliance rates across specialties, with trauma surgeons demonstrating a higher non-compliance rate than other surgical groups (χ² = 9.62, p = 0.002). This suggests that specialty-specific practices may influence adherence to thyroid protection protocols (Figure 1).

Breakdown by role

**Figure 2:**
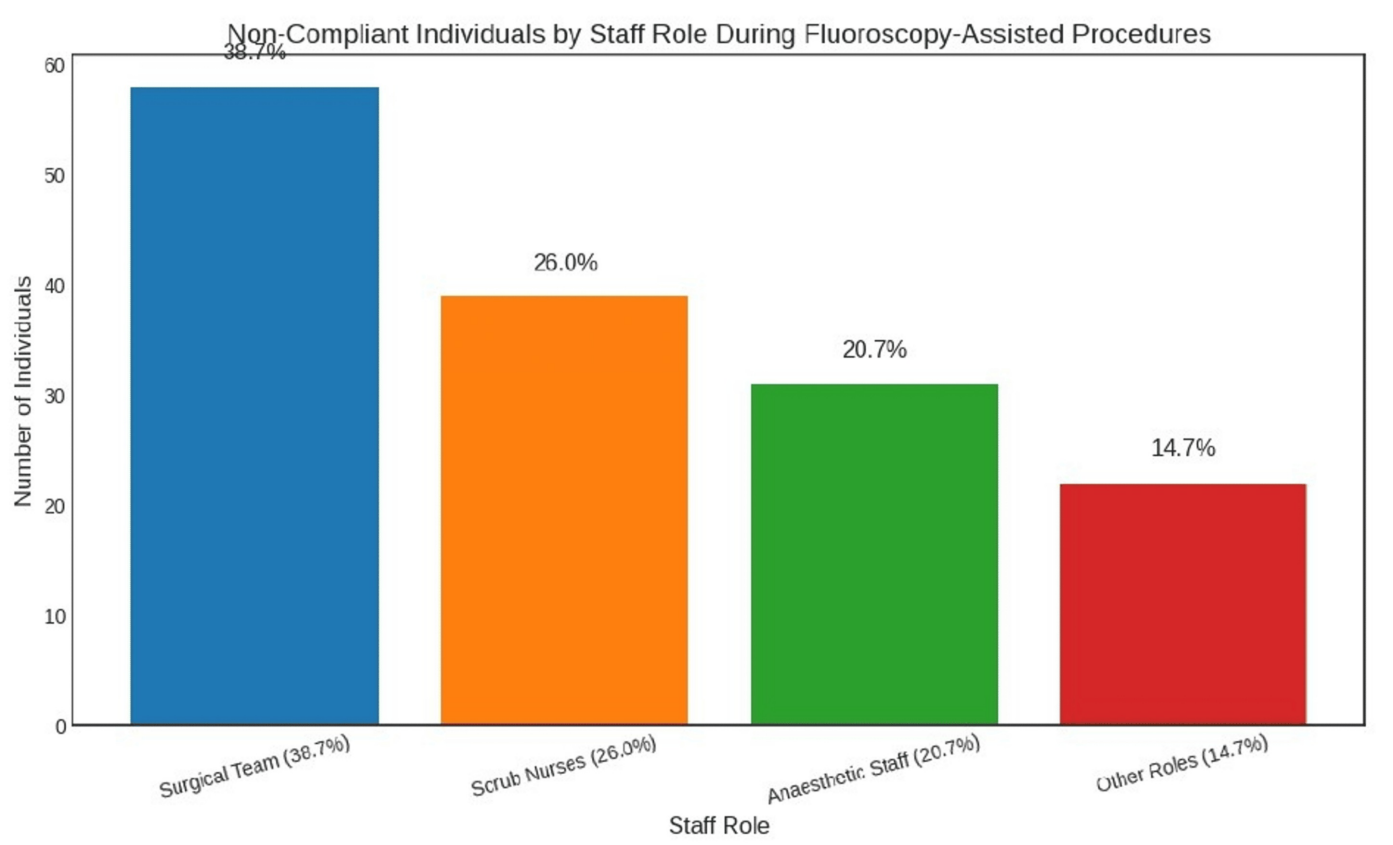
Non-compliance rate based on staff role

Within the same group of 150 non-compliant individuals (no thyroid protection), 58 (38.7%) were surgical team members, 39 (26.0%) were scrub nurses, 31 (20.7%) were anaesthetic staff, and 22 (14.7%) belonged to other roles (Figure 2). Chi-square analysis revealed significantly higher non-compliance among surgical team members compared to other roles (χ² = 14.87, p < 0.001, OR = 2.31, 95% CI: 1.52-3.52). Scrub nurses also showed elevated non-compliance (χ² = 7.43, p = 0.006, OR = 1.74, 95% CI: 1.18-2.89), as did anaesthetic staff (χ² = 5.12, p = 0.024, OR = 1.58, 95% CI: 1.06-2.52), and individuals from other roles (χ² = 3.87, p = 0.049, OR = 1.42, 95% CI: 1.01-2.21).

Among the 712 staff members assessed across 91 operations, only seven (0.98%) were completely non-compliant with PPE protocols, wearing neither a thyroid shield nor a lead gown. A chi-square test comparing these seven entirely non-compliant staff to the 705 who wore at least one protective item yielded a highly significant result (χ² = 684.28, p < 0.0001), indicating that complete non-compliance was far less common than expected by chance. These findings highlight strong overall adherence to PPE protocols among the staff.

## Discussion

This audit highlights a concerning disparity in radiation protection compliance among operating theatre staff. While overall adherence to safety protocols during fluoroscopy-assisted procedures was relatively high, a significant minority, particularly among trauma surgeons and surgical team members, did not comply. The presence of individuals operating without any protective equipment underscores the urgent need for systemic improvements in training, equipment availability, and accountability.

C-arm fluoroscopy plays a crucial role in the diagnosis and treatment of trauma and orthopaedic operations. It enables precise implant placement for accurate fracture fixation by providing continuous real-time X-ray imaging [3,10]. However, the growing reliance on C-arm fluoroscopy in orthopaedic procedures has led to increased exposure to ionising radiation. Surgeons often operate near the patient and the X-ray source, making it challenging to avoid scatter radiation [11,12]. As a result, minimising radiation exposure during orthopaedic surgery is essential [13].

Ionising radiation exposure in clinical settings typically arises from three main sources: direct contact with the primary X-ray beam, scatter radiation emitted from the patient or operating table, and minor leakage from the X-ray tube [14]. Direct exposure to the primary beam poses little risk unless the operator’s hand enters the beam’s path during the procedure. Similarly, leakage radiation is minimal and generally not a significant concern for practitioners. The primary hazard for healthcare professionals, especially those performing procedures, is scatter radiation. This type of radiation is generated when the primary beam interacts with the patient’s body, dispersing in multiple directions. Consequently, the proximity of the operator to the patient is the most critical factor influencing radiation exposure [15,16].

Radiation exposure in theatre settings is not uniform and varies based on several clinical and operational factors. More complex fractures require longer imaging times, while longer implants and additional hardware increase exposure. Surgeon experience also plays a role, with less experienced surgeons tending to rely more heavily on fluoroscopy. Additionally, larger patients necessitate higher radiation doses to achieve adequate image clarity [5]. The thyroid gland is particularly vulnerable, with a tissue-weighting factor of 0.04. In this audit, the average thyroid dose measured during femoral intramedullary nailing was 142 microsieverts (μSv) (0.142 mSv), corresponding to an effective dose of 0.00568 mSv. Although this is below occupational limits, cumulative exposure, particularly in high-volume trauma, can pose significant long-term risks. The equivalent dose for the thyroid, calculated as 1 Gy × 1 × 1 = 1 Sv, further illustrates its sensitivity to ionising radiation.

Compliance with PPE protocols is multifactorial, influenced by individual behaviour, access to appropriate equipment, and the prevailing workplace culture. Quantitative analysis enabled targeted identification of specific roles and specialities requiring intervention, allowing for more focused educational and operational strategies. Alarmingly, the audit also observed radiographers using mobile phones during procedures, which compromises both procedural efficiency and radiation safety.

To address these gaps, a multifaceted approach is essential. Educational interventions must be paired with equipment audits and cultural shifts that prioritise safety. The appointment of radiation protection supervisors and consultation with radiation protection advisors are mandated under IRR17 regulations and play a critical role in governance. Radiation exposure must adhere to the "as low as reasonably achievable" (ALARA) principle and remain ALARA. This can be accomplished by applying core radiation safety practices, incorporating protective engineering and design measures, and ensuring the use of appropriate PPE [17]. Sustained improvement depends on regular audits, feedback mechanisms, and visible leadership engagement [7,8].

Strengths

This audit benefited from a large sample size and detailed role-specific analysis, enhancing the reliability and relevance of its findings. The integration of observational data with checklist-based assessments provided a robust framework for evaluating compliance and identifying actionable areas for improvement. A key strength of the methodology was its blinded design; no operating theatre staff, apart from the radiographers, were aware that radiation protection behaviour was being monitored. This approach minimised observer bias and ensured that the data reflected genuine staff conduct, particularly regarding the use (or absence) of radiation protection during fluoroscopy. As a result, the audit offers a more accurate representation of routine practice and highlights areas where targeted interventions may be most needed.

Limitations

As a single-centre study, the generalisability of findings to other institutions is limited. Observational bias may have influenced the accuracy of recorded behaviours, and the audit did not establish a direct correlation between PPE compliance and actual radiation dose outcomes.

Recommendations to improve compliance

Targeted workshops were conducted to engage theatre teams and reinforce best practices, with an emphasis on shared responsibility for radiation safety. An equipment audit was carried out to ensure the availability of thyroid shields and dosimeters, particularly for surgical staff. Finally, a follow-up audit and staff survey were planned to evaluate the effectiveness of these interventions and guide future improvements.

This audit revealed that while many theatre staff adhered to radiation protection protocols, a significant minority did not, particularly among surgeons and surgical team members. The presence of individuals without any protective equipment emphasises the need for systemic improvements in training, equipment availability, and accountability.

## Conclusions

This audit underscores that while radiation protection compliance during fluoroscopy-assisted procedures is generally high, notable gaps persist, particularly among trauma surgeons and surgical team members. These disparities reflect a complex interplay of behavioural, cultural, and operational factors that influence PPE use and safety practices.

Given the thyroid gland's heightened sensitivity and the cumulative risks associated with repeated exposure, consistent adherence to protective protocols is critical. Addressing these gaps requires a multifaceted strategy that combines targeted education, equipment audits, regulatory oversight, and visible leadership engagement. Sustained improvement will depend on fostering a culture of shared responsibility and continuous evaluation within the operating theatre.
